# Reply to: Awake prone positioning for non-intubated patients with COVID-19-related acute hypoxic respiratory failure: a systematic review based on eight high-quality randomized controlled trials

**DOI:** 10.1186/s12879-023-08834-4

**Published:** 2023-11-23

**Authors:** Xinfang Xie, Youwei Lin

**Affiliations:** Department of Intensive Care, Yuhuan Second People’s Hospital of Health community Group, Huanbaozhong Road 77#, Taizhou, Zhejiang China

**Keywords:** Prone positioning, COVID-19, Mortality

Dear Editor,

In a recent meta-analysis [1], Dr.Cao et al. investigated the efficacy of awake-prone positioning versus usual care in hypoxemic COVID-19 patients in medical wards. A total of eight trials were included. The authors reported that awake-prone positioning is safe and feasible in non-intubated patients with AHRF caused by COVID-19 and can significantly reduce the intubation rate. We want to add some comments.

First, a literature search was conducted in PubMed, Web of Science, Cochrane, Embase, and Scopus databases, from December 1, 2019 to November 1, 2022. However, one trial [2] seem to be missing, which was also randomized and investigated the potential efficacy of awake-prone positioning in COVID-19. Therefore, these should be included to avoid selection bias.

Second, the efficacy of prone positioning in COVID-19 has been investigated in dozens of studies [3]. The major conclusions of the current study were that awake prone positioning can significantly reduce the intubation rate, but showed no significant benefit in mortality. We suggest this result should be interpreted with caution. Substantial evidence indicates that intubation was associated with severe disease condition, which is a major risk factor for high mortality. Therefore, to a certain extent, reducing the intubation rate can reduce the mortality rate. In the current study, although not significant, a beneficial trend in decreasing mortality was also observed (odds ratio 0.88, 95%CI 0.72–1.08). Therefore, whether this non-significant result was influenced by an insufficient sample size remains uncertain. Trial sequence analysis [4] is an option to determine whether the current sample size for mortality reaches the threshold of statistical significance. In a previous analysis including 174 meta-analyses, TSA (30% relative risk reduction) showed that almost 80% of ninty-five statistically nonsignificant meta-analyses had insufficient information size and showed potentially false positive results.

## Data Availability

Not available.

## References

[1] Cao W, He N, Luo Y, Zhang Z (2023). Awake prone positioning for non-intubated patients with COVID-19-related acute hypoxic Respiratory Failure: a systematic review based on eight high-quality randomized controlled trials. BMC Infect Dis.

[2] Taylor SP, Bundy H, Smith WM, Skavroneck S, Taylor B, Kowalkowski MA (2021). Awake Prone Positioning Strategy for Nonintubated hypoxic patients with COVID-19: a pilot trial with embedded implementation evaluation. Ann Am Thorac Soc.

[3] De Bels D, Redant S, Honore PM (2021). Prone positioning in Coronavirus Disease 2019 patients with Acute Respiratory Distress Syndrome: how and when is the best way to do it?. J Transl Int Med.

[4] Brok J, Thorlund K, Gluud C, Wetterslev J (2008). Trial sequential analysis reveals insufficient information size and potentially false positive results in many meta-analyses. J Clin Epidemiol.

